# Micronutrient deficiencies and new-onset atrial fibrillation in a community-based cohort: data from PREVEND

**DOI:** 10.1007/s00392-023-02276-3

**Published:** 2023-08-17

**Authors:** Ali A. Al-Mubarak, Niels Grote Beverborg, Victor Zwartkruis, Colinda van Deutekom, Martin H. de Borst, Ron T. Gansevoort, Stephan J. L. Bakker, Daan J. Touw, Rudolf A. de Boer, Peter van der Meer, Michiel Rienstra, Nils Bomer

**Affiliations:** 1 Department of Cardiology, University of Groningen, University Medical Center Groningen, UMCG Post-Zone AB43, PO Box 30.001, 9700 RB Groningen, The Netherlands; 2 Division of Nephrology, Department of Internal Medicine, University Medical Center Groningen, University of Groningen, Groningen, The Netherlands; 3 Department of Clinical Pharmacy and Pharmacology, University of Groningen, University Medical Center Groningen, Groningen, The Netherlands; 4 Department of Cardiology, Erasmus University Rotterdam, Erasmus University Medical Center, Rotterdam, The Netherlands

**Keywords:** Selenium, Iron, Magnesium, Micronutrients, Malnutrition, Atrial fibrillation

## Abstract

**Aim:**

Malnutrition has been linked to cardiovascular diseases. Both selenium and iron deficiency have been associated with worse prognosis in patients with heart failure (HF). Yet, little is known about the role of micronutrients in the development of atrial fibrillation (AFib). In this study, we aimed to elucidate the association of micronutrient deficiencies with new-onset AFib.

**Methods:**

Selenium, magnesium, and iron parameters were measured in a well-characterized prospective cohort study (*N* = 5452). Selenium deficiency was defined as serum selenium < 70 μg/L, iron deficiency as serum ferritin < 30 μg/L, and magnesium deficiency as plasma magnesium < 0.85 mmol/L. New-onset AFib was the primary outcome. Additionally, we tested for previously reported effect-modifiers where applicable.

**Results:**

Selenium, iron, and magnesium deficiency was observed in 1155 (21.2%), 797 (14.6%), and 3600 (66.0%) participants, respectively. During a mean follow-up of 6.2 years, 136 (2.5%) participants developed new-onset AFib. Smoking status significantly interacted with selenium deficiency on outcome (*p* = 0.079). After multivariable adjustment for components of the CHARGE-AF model, selenium deficiency was associated with new-onset AFib in non-smokers (HR 1.69, 95% CI 1.09–2.64, *p* = 0.020), but not in smokers (HR 0.78, 95% CI 0.29–2.08, *p* = 0.619). Magnesium deficiency (HR 1.40, 95% CI 0.93–2.10, *p* = 0.110) and iron deficiency (HR 0.62, 95% CI 0.25–1.54, *p* = 0.307) were not significantly associated with new-onset AFib.

**Conclusion:**

Selenium deficiency was associated with new-onset AFib in non-smoking participants. Interventional studies that investigate the effects of optimizing micronutrients status in a population at risk are needed to assess causality, especially in those with selenium deficiency.

**Graphical abstract:**

Micronutrients deficiencies (selenium, iron, and magnesium) have been associated with cardiovascular diseases and mitochondrial dysfunction in human cardiomyocytes. However, it is not known whether these deficiencies are associated with atrial fibrillation. To investigate this question, we measured all three micronutrients in 5452 apparently healthy individuals. After a mean follow-up of 6.2 years, there were 136 participants who developed atrial fibrillation. Participants with selenium deficiency had a significant increased risk to develop atrial fibrillation, as did the participants with two or more deficiencies.

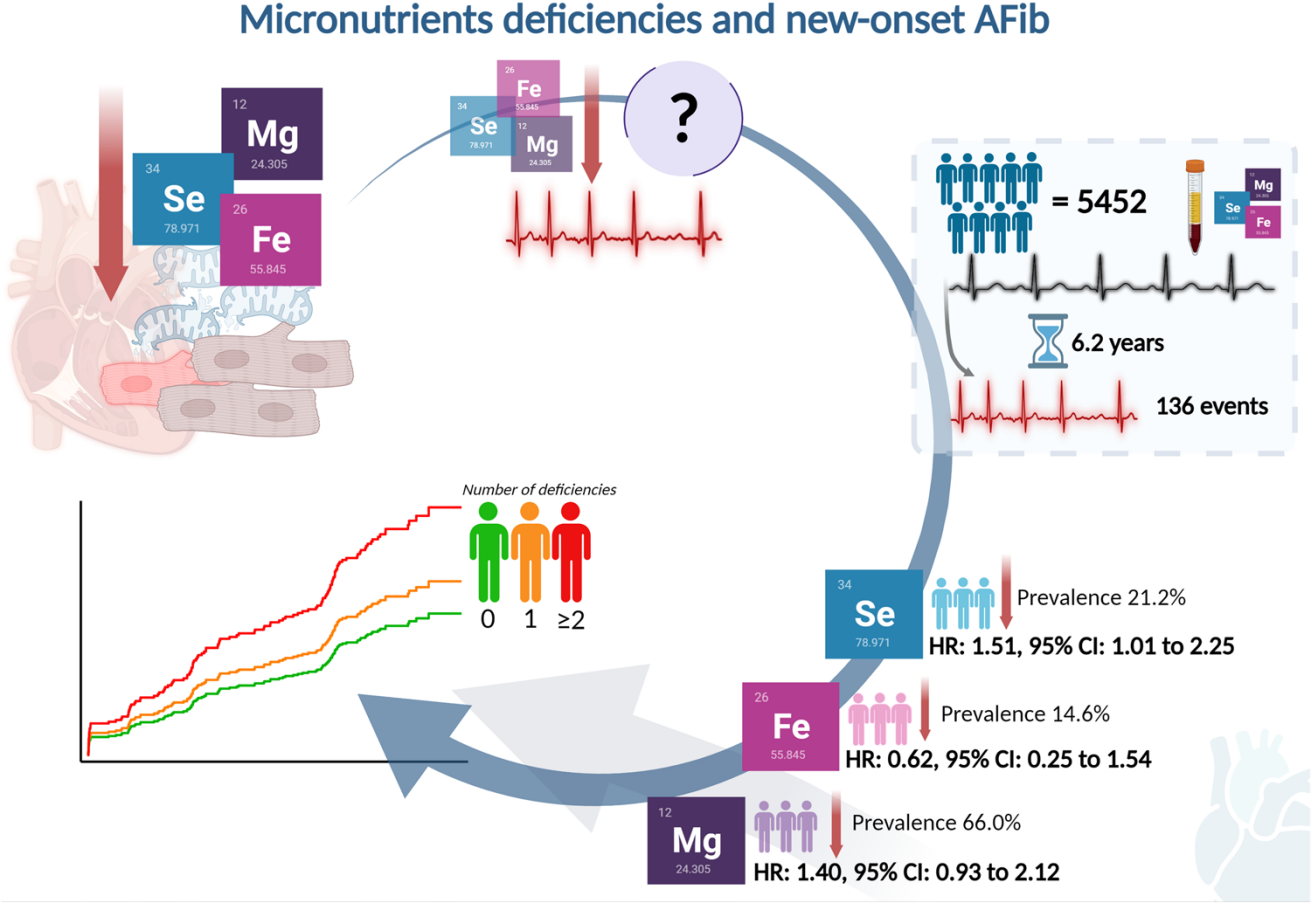

**Supplementary Information:**

The online version contains supplementary material available at 10.1007/s00392-023-02276-3.

## Introduction

Atrial fibrillation (AFib) is a highly prevalent cardiovascular disease with increasing incidence worldwide. AFib leads to various serious sequelae, such as stroke, myocardial infarction, and heart failure (HF) [1]. Several modifiable risk factors are associated with AFib, including diet-related risk factors such as hypertension, diabetes, and obesity [1]. Individuals with high cardiovascular risk factor profile have higher lifetime risk of AFib [2], suggesting that early intervention and control of modifiable risk factors may reduce incident AFib.

Epidemiological data of specific dietary factors in relation to new-onset AFib are limited and mainly based on dietary questionnaires that are less suitable to provide objective measurements of specific elements of the diet, especially micronutrients [3, 4]. Nevertheless, current evidence indicates potential roles for nutrient-related factors in the development of AFib, as supplementations with magnesium, vitamin C, and polyunsaturated fatty acids (i.e., antioxidant supplementations) showed beneficial effects in clinical settings [1, 5].

Deficiencies of micronutrients (e.g., magnesium, iron, selenium, and iodine) are common worldwide and occur at all levels of the population, regardless of the weight status (i.e., obese or individuals with healthy weight) [6]. They are associated with cardiovascular diseases, including stroke, hypertension, coronary artery disease, and heart failure (HF) [7, 8]. AFib and HF are conditions that are closely interconnected, share risk factors and pathophysiological processes [9]. The significance of several micronutrients is established in HF [8], but little is known for AFib. As an example, treating iron deficiency (ID) is recommended in recently published guidelines as it improved outcome in patients with HF [8]. In addition, Se deficiency (SeD) strongly is associated with worse exercise capacity and worse prognosis in patients with HF, as well as with new-onset HF in the general population [10, 11].

Furthermore, SeD and ID share similar predicators in patients with HF [12] and both are involved in several essential cellular processes, including redox homeostasis, modulating inflammatory response as well as maintaining normal mitochondrial function in human cardiomyocytes [8, 10, 13]. Since AFib is a condition that often coexists with HF, one can hypothesize that SeD and ID may associate with AF. However, evidence about the association between SeD and ID with new-onset AF is lacking.

In addition, magnesium is a micronutrient known to modulate the electrical activity of cardiomyocytes [14]. Low plasma concentrations of magnesium were moderately associated with new-onset AFib in the US [15] and administering magnesium to patients with AFib was likely to improve the management of acute AFib episode [1, 16]. Whether these observations are similar for other minerals and for different populations is not known. In this study, we aim to assess the association of SeD, ID, and MgD with new-onset AFib in a well-characterized, prospective, general population cohort. This study will provide insights to identify additional, diet-specific, modifiable factors that relate to new-onset AFib.

## Materials and methods

### Study population

Samples and data from the PREVEND (Prevention of Renal and Vascular ENd-stage Disease) cohort study were utilized. The study has been described in detail elsewhere [17]. In summary, the inhabitants of the city of Groningen in the Netherlands were asked to complete a questionnaire and provide a morning urine sample. Pregnant women and patients with diabetes mellitus type 1 were excluded. After analyzing urinary albumin excretion, 8592 participants were included, 6000 participants with urinary albumin excretion ≥ 10 mg/L and 2592 participants with urinary albumin excretion < 10 mg/L. Starting from 1997, the participants followed up at 3-year intervals. On each visit, a fasting venous blood sample was collected and stored at − 80 °C. In addition, a 12-lead ECG was taken at each visit. Participants with prevalent AFib (*N* = 84) or unknown rhythm status (*N* = 178) were excluded. Micronutrients were measured in samples from the second visit, which was attended by 6894 participants. Therefore, the second PREVEND visit was considered as the baseline for the present analyses. In the current analysis, we included all participants with available measurements of selenium, magnesium as well as iron parameters (*N* = 5452) (Online Fig. 1). Serum selenium was measured using inductively coupled plasma mass spectrometry as described elsewhere [10]. Iron parameters were measured using a colorimetric assay, immunoassay, and immunoturbidimetric assay as outlined before [18]. Total plasma magnesium was measured using the Roche modular assay as described previously [19]. All samples were collected in the morning and all measurements were performed in one central laboratory, using standardized diagnostic tools utilized in daily clinical practice. The low limits of detections were 20 μg/L, 0.5 μg/L, and 0.1 mmol/L for selenium, ferritin, and magnesium concentrations, respectively. The study was performed in line with the principles of the declaration of Helsinki and was approved by the local Medical Ethics Committee. All participants provided written informed consent.

### Definitions

SeD was defined as a serum selenium concentration < 70 μg/L [10]. Since the studied population consisted of apparently healthy individuals with low likelihood for active infection, participants with serum ferritin levels < 30 μg/L were considered to have ID [20, 21]. A commonly used cut-off for MgD is < 0.75 mmol/L, which is based on the distribution in a normal population [22]. However, given the complexity of magnesium metabolism, the definition of MgD is not well-established. When considering adverse health outcomes (i.e., CVD morbidity and mortality) in addition to the risk of chronic latent MgD (i.e., individuals with magnesium concentrations 0.75–0.85 mmol/L and a positive magnesium loading test), Costello et al. suggested a cut-off of < 0.85 mmol/L. We subsequently chose for the latter cutoff for the primary analysis and the former cutoff as a sensitivity analysis [22]. SeD and ID were selected for this investigation because of their relevance in the context of HF, which, in turn, is closely related to AFib and share several pathophysiological mechanisms, while MgD was selected because of its established relevance in cardiac electrophysiology.

Furthermore, anemia was defined as hemoglobin level < 12 g/dL in women and < 13 g/dL in men. Type 2 diabetes was considered present if participants had either (1) fasting blood glucose > 7.0 mmol/L, (2) non-fasting blood glucose > 11.1 mmol/L, (3) documented use of antidiabetic medication, or (4) if participants reported that they had physician-diagnosed diabetes. In addition, we defined the smoking status as either current or no current smokers. Kidney function was calculated based on CKD-EPI creatinine equation.

### Incidence of atrial fibrillation

The details about the ascertainment of incident AFib have been described elsewhere [23]. In summary, the diagnosis was made if either atrial flutter or AFib was observed (1) on a 12-lead ECG obtained during the follow-up visits of the PREVEND study, or (2) during a recorded hospital visit in one of the two hospitals of the city of Groningen. All ECGs were digitally stored and screened electronically for atrial flutter, ectopic atrial rhythm, or the absence of PR interval. Afterward, two independent observers (i.e., physicians with experience in evaluating ECG’s) reviewed all suspected AFib cases as determined by electronic screening. When there was inconsistency between the observers or when both observers agreed on the diagnosis of atrial flutter or AFib, two independent cardiologists additionally validated each case. The date of the first ECG with confirmed AFib was considered the date of incident AFib.

### Statistical analysis

Normally distributed data was reported as mean (standard deviation (SD)), non-normally distributed data as median (interquartile range (IQR)), and binary variables as frequencies (percentages). To compare the baseline characteristics, we used the independent samples *t* test, Wilcoxon rank-sum test, or Pearson’s chi-squared test where appropriate. Stepwise multivariable logistic regression analyses were performed for the potential associates of the participant characteristics with ID and MgD (Online Table S1–2). Associates of selenium were previously reported [11]. Our primary outcome was new-onset AFib. The current analysis is a retrospective analysis of prospectively assessed data. To evaluate the association of SeD, ID, and MgD with new-onset AFib, Cox proportional hazards regression analyses were performed. We evaluated each deficiency separated, as well as the deficiencies combined in three categories: participants with no deficiency, one deficiency, and two or more deficiencies in order to evaluate the potential cumulative effects as each deficiency may have consequences to different biological processes [13, 14, 24]. In addition, as a sensitivity analyses, competing risk regression analyses were performed with death as a competing risk. Proportionality assumptions were assessed using Grambsch–Therneau test and by assessing Schoenfeld residuals, which showed no violations of the assumptions. The association between micronutrient deficiencies and new-onset AF was assessed in a uni-variable model (i.e., model A) and two multivariable models (i.e., model B and model C). Model B was adjusted for the associates of the studied deficiencies which we considered to be confounders since most of them differed between the participants based on their deficiency status and are directly or indirectly related to the deficiencies and the outcome [12, 14, 24]. This model B included the following variables: age, sex, BMI, smoking status, glucose, total cholesterol, anemia, kidney function, calcium, potassium, and CRP concentrations as well as the three deficiencies. Model C was adjusted for components of the CHARGE-AF risk model (age, smoking status, weight, height, systolic blood pressure, the use of antihypertensive drugs, diabetes mellitus, prevalent heart failure, and history of myocardial infarction) [25]. All models were adjusted for 24-h urinary albumin excretion in order to correct for study design. In a secondary analysis, we excluded participants with prevalent HF and adjusted for interim HF events. All analyses were conducted as complete case analysis (Online Table S3). Because of the previously reported interaction with smoking and because of the frequently reported low selenium status in smokers [26, 27], as planned analyses, we tested for interaction between SeD and smoking in relation to new-onset AFib. Additionally, we tested for interactions between sex and each deficiency as exploratory analyses. As an extra sensitivity analysis, we investigated the association between each deficiency and new-onset AFib in participants older than 60 and older than 70. *p* values < 0.1 and < 0.05 were considered significant for the interaction term and all other analyses, respectively. In order to visualize our findings, we developed contour plots, stratified by the found effect-modifiers, as well as restricted cubic splines of the continuous variables with four knots based on Harrell’s recommended percentiles. We also constructed Venn diagram to visualize the overlap between the three deficiencies. Stata version 16.0 and R version 4.1.0 were used to perform all analyses.

## Results

### Participant characteristics and AFib incidence

**Fig. 1:**
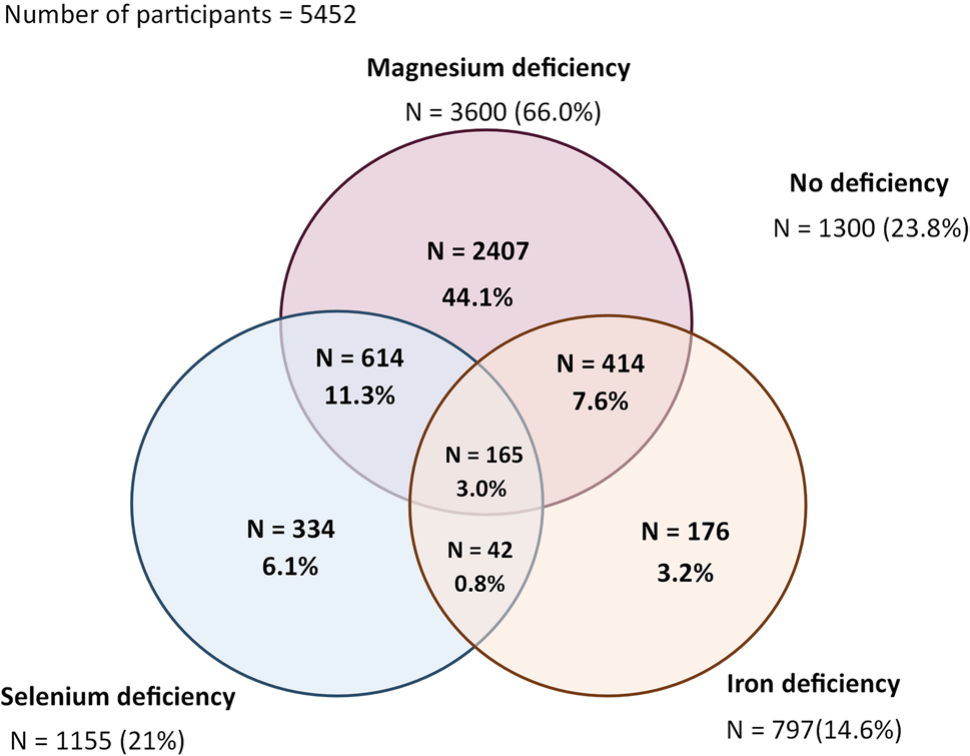
Venn diagram. The numbers reported outside each circle indicate the numbers of each deficiency in the total cohort. The overlapping areas indicate the number of participants with two or three deficiencies

The measurements of selenium, iron, and magnesium were available for 5452 participants. The mean age was 53.3 (12.0) years and 2843 (52.1%) of all participants were women. In total, 1155 (21.2%) participants had SeD, 797 (14.6%) had ID and MgD was present in 3600 (66.0%) participants as defined by plasma magnesium < 0.85 mmol/L (vs. 437 (8.0%) participants as defined by plasma magnesium < 0.75 mmol/L) (Fig. 1).

Mean selenium concentration for the SeD group was 60.7 (7.5) μg/L, while the non-deficient group had a mean concentration of 90.8 (16.1) μg/L. Compared to participants without SeD, participants with SeD were more likely to smoke (368 (32.1%) vs. 1133 (26.6%), *p* < 0.001), more likely to have a history of myocardial infarction (92 (8.1%) vs. 241 (5.7%), *p* = 0.006), more likely to have anemia (144 (12.5%) vs. 370 (8.7%), *p* < 0.001) or ID (207 (17.9%) vs. 590 (13.7%), *p* < 0.001), and had higher levels of C-reactive protein (CRP) (1.5 (0.7, 3.4) mg/L vs. 1.3 (0.6, 2.8), *p* < 0.001) (Table 1). No differences were found in the distribution for age, sex, BMI, and history of diabetes.

**Table 1:** Baseline characteristics

	All participants	No Selenium deficiency	Selenium deficiency	*p* value	No Magnesium deficiency (> 0.85 mmol/L)	Magnesium deficiency (≤ 0.85 mmol/L)	*p* value	No iron deficiency	Iron deficiency	*p* value
*N*	5452	4297	1155		1852	3600		4655	797	
Serum selenium (µg/L)	84.5 (19.2)	90.8 (16.1)	60.7 (7.5)	< 0.001	85.1 (19.2)	84.2 (19.2)	0.093	85.0 (19.3)	81.1 (18.4)	< 0.001
Plasma magnesium (mmol/L)	0.82 (0.06)	0.82 (0.07)	0.82 (0.06)	0.1	0.89 (0.06)	0.79 (0.04)	< 0.001	0.83 (0.07)	0.81 (0.06)	< 0.001
Ferritin (µg/L)	96.0 (47.5, 172.0)	99.0 (50.0, 175.0)	86.0 (41.0, 164.0)	< 0.001	102.5 (54.0, 177.5)	93.0 (44.0, 169.0)	< 0.001	114.0 (67.0, 190.0)	17.0 (10.0, 24.0)	< 0.001
Age (years)	53.3 (12.0)	53.4 (11.9)	52.9 (12.2)	0.22	53.8 (12.0)	53.0 (11.9)	0.025	54.4 (11.9)	46.9 (10.1)	< 0.001
Women (%)	2843 (52.1%)	2223 (51.7%)	620 (53.7%)	0.24	894 (48.3%)	1949 (54.1%)	< 0.001	2151 (46.2%)	692 (86.8%)	< 0.001
Body Mass Index (kg/m^2^)	26.6 (4.3)	26.6 (4.2)	26.7 (4.4)	0.4	26.4 (4.1)	26.7 (4.4)	0.013	26.9 (4.3)	25.3 (4.2)	< 0.001
Systolic blood pressure (mmHg)	125.6 (18.7)	125.6 (18.8)	125.9 (18.3)	0.62	126.5 (19.2)	125.2 (18.4)	0.017	127.0 (18.9)	117.8 (15.7)	< 0.001
Current smoker (%)	1501 (27.8%)	1133 (26.6%)	368 (32.1%)	< 0.001	495 (27.0%)	1006 (28.2%)	0.35	1312 (28.4%)	189 (24.0%)	0.01
History of Myocardial infarction (%)	333 (6.2%)	241 (5.7%)	92 (8.1%)	0.003	109 (6.0%)	224 (6.3%)	0.62	296 (6.5%)	37 (4.7%)	0.057
History of diabetes (%)	317 (5.9%)	261 (6.2%)	56 (5.0%)	0.13	53 (2.9%)	264 (7.5%)	< 0.001	298 (6.5%)	19 (2.4%)	< 0.001
History of CVA (%)	60 (1.1%)	42 (1.0%)	18 (1.6%)	0.092	18 (1.0%)	42 (1.2%)	0.5	52 (1.1%)	8 (1.0%)	0.76
History of HF (%)	47 (0.9%)	34 (0.8%)	13 (1.1%)	0.28	13 (0.7%)	34 (0.9%)	0.36	43 (0.9%)	4 (0.5%)	0.23
Glucose (mmol/L)	5.0 (1.2)	5.0 (1.2)	5.0 (1.1)	0.033	4.9 (0.8)	5.1 (1.3)	< 0.001	5.1 (1.2)	4.7 (0.9)	< 0.001
Cholesterol (mmol/L)	5.4 (1.1)	5.5 (1.1)	5.3 (1.1)	< 0.001	5.5 (1.1)	5.4 (1.0)	0.001	5.5 (1.1)	5.1 (1.0)	< 0.001
eGFR (mL/min/1.73 m^2^)	92.2 (16.8)	92.2 (16.7)	92.0 (17.4)	0.72	90.9 (16.8)	92.8 (16.8)	< 0.001	91.2 (16.9)	97.7 (15.2)	< 0.001
Kidney function below 60 ml/min/m^2^ (%)	209 (4.0%)	161 (3.9%)	48 (4.5%)	0.39	77 (4.4%)	132 (3.8%)	0.36	197 (4.5%)	12 (1.6%)	< 0.001
Urinary albumin excretion (mg/24 h)	8.0 (5.9, 13.0)	8.0 (5.8, 13.1)	7.9 (6.0, 12.8)	0.58	8.0 (5.9, 12.2)	8.0 (5.9, 13.5)	0.41	8.1 (6.0, 13.5)	7.3 (5.5, 11.2)	< 0.001
Hemoglobin (mmol/L)	8.5 (0.8)	8.5 (0.7)	8.5 (0.8)	0.054	8.6 (0.8)	8.5 (0.7)	< 0.001	8.6 (0.7)	7.8 (0.7)	< 0.001
Anemia (%)	514 (9.5%)	370 (8.7%)	144 (12.5%)	< 0.001	158 (8.6%)	356 (9.9%)	0.1	275 (5.9%)	239 (30.1%)	< 0.001
MCV (fL)	90.5 (4.5)	90.5 (4.5)	90.3 (4.8)	0.16	90.4 (4.4)	90.5 (4.6)	0.28	91.0 (4.1)	87.8 (5.7)	< 0.001
Hematocrit (v/v)	0.4 (0.0)	0.4 (0.0)	0.4 (0.0)	0.032	0.4 (0.0)	0.4 (0.0)	< 0.001	0.4 (0.0)	0.4 (0.0)	< 0.001
Serum iron (umol/L)	15.9 (5.7)	16.0 (5.6)	15.3 (5.7)	< 0.001	16.0 (5.5)	15.8 (5.7)	0.3	16.4 (5.4)	13.0 (6.4)	< 0.001
Transferrin (g/L)	2.6 (0.4)	2.6 (0.4)	2.6 (0.4)	0.91	2.6 (0.4)	2.6 (0.4)	0.027	2.5 (0.4)	2.9 (0.5)	< 0.001
Transferrin saturation (%)	151.2 (71.9, 280.3)	154.5 (75.5, 284.8)	140.0 (60.4, 264.1)	< 0.001	161.8 (82.7, 287.8)	146.5 (67.7, 276.9)	0.002	182.7 (104.1, 310.1)	23.1 (13.8, 32.7)	< 0.001
Iron deficiency (%)	797 (14.6%)	590 (13.7%)	207 (17.9%)	< 0.001	218 (11.8%)	579 (16.1%)	< 0.001	0	797 (14.6%)	
Selenium deficiency (%)	1155 (21.2%)	0	1155 (21.2%)		376 (20.3%)	779 (21.6%)	0.25	948 (20.4%)	207 (26.0%)	< 0.001
Plasma calcium (mmol/L)	2.3 (0.1)	2.3 (0.1)	2.3 (0.1)	0.29	2.3 (0.1)	2.3 (0.1)	0.013	2.3 (0.1)	2.3 (0.1)	< 0.001
Plasma potassium (mmol/L)	4.2 (0.3)	4.2 (0.3)	4.2 (0.3)	0.27	4.3 (0.3)	4.2 (0.3)	< 0.001	4.2 (0.3)	4.2 (0.3)	< 0.001
hs-CRP (mg/L)	1.3 (0.6, 2.9)	1.3 (0.6, 2.8)	1.5 (0.7, 3.4)	< 0.001	1.3 (0.6, 2.9)	1.3 (0.6, 3.0)	0.98	1.4 (0.7, 3.1)	0.9 (0.4, 2.2)	< 0.001
Antihypertensive medication	1176 (21.6%)	914 (21.3%)	262 (22.8%)	0.27	359 (19.4%)	817 (22.7%)	0.006	1084 (23.3%)	92 (11.5%)	< 0.001

In contrast to participants with SeD, participants with ID had a profile that appeared to be more healthy (Table 1). Compared to participants without ID, those with ID were younger (46.9 (10.1) years old vs. 54.4 (11.9), *p* < 0.001), more likely to be women (692 (86.8%) vs. 2151 (46.2%), *p* < 0.001), and had lower BMI (25.3 (4.2) (kg/m^2^) vs. 26.9 (4.3), *p* < 0.001) and lower systolic blood pressure (117.8 (15.7) mmHg vs. 127.0 (18.9), *p* < 0.001). They were less likely to smoke (189 (24.0%) vs. 1312 (28.4%), *p* < 0.001), less likely to have a history of myocardial infarction (37 (4.7%) vs. 296 (6.5%), *p* = 0.057), they had a lower prevalence of diabetes (19 (2.4%) vs. 298 (6.5%), *p* < 0.001), lower levels of glucose (4.7 (0.9) mmol/L vs. 5.1 (1.2), *p* < 0.001), and lower total cholesterol (5.1 (1.0) mmol/L vs. 5.5 (1.1), *p* < 0.001). However, they were more likely to have anemia (239 (30.1%) vs. 275 (5.9%), *p* < 0.001) and SeD (207 (26.0%) vs. 948 (20.4%), *p* < 0.001).

Furthermore, mean magnesium concentration in the total cohort was 0.82 mmol/L. Participants with MgD were more likely to be women (1949 (54.1%) vs. 894 (48.3%), *p* < 0.001), have diabetes (264 (7.5%) vs. 53 (2.9%), *p* < 0.001), have anemia (356 (9.9%) vs. 158 (8.6%), *p* = 0.10), and have ID (579 (16.1%) vs. 218 (11.8%), *p* < 0.001). Additionally, they had higher use of antihypertensive medications (817 (22.7%) vs. 359 (19.4%), *p* = 0.006) and higher BMI (26.7 (4.4) kg/m^2^ vs. 26.4 (4.1), *p* = 0.013) (Table 1). Similar patterns were observed using the secondary cut-off for MgD (Online Table S4). Additionally, based on the secondary cut-off, participants with MgD had higher prevalence of cardiovascular diseases (i.e., myocardial infarction, CVA, and HF), lower eGFR and higher hs-CRP concentrations. Selenium status didn’t differ significantly between participants with or without MgD, regardless of the cut-off.

There were 136 (2.5%) AFib events that occurred within a mean follow-up period of 6.2 years (Incidence rate of 4.02 per 1000 person-years). Men had 97 new-onset AFib events and non-smoking participants had 102 events. Of the participants who developed AFib, 25.7% had SeD, 5.1% had ID, and 75% had MgD compared to 21.1%, 14.9%, and 65.8% in those who did not develop AFib, respectively.

### Selenium deficiency is associated with new-onset atrial fibrillation (in non-smokers)

**Fig. 2:**
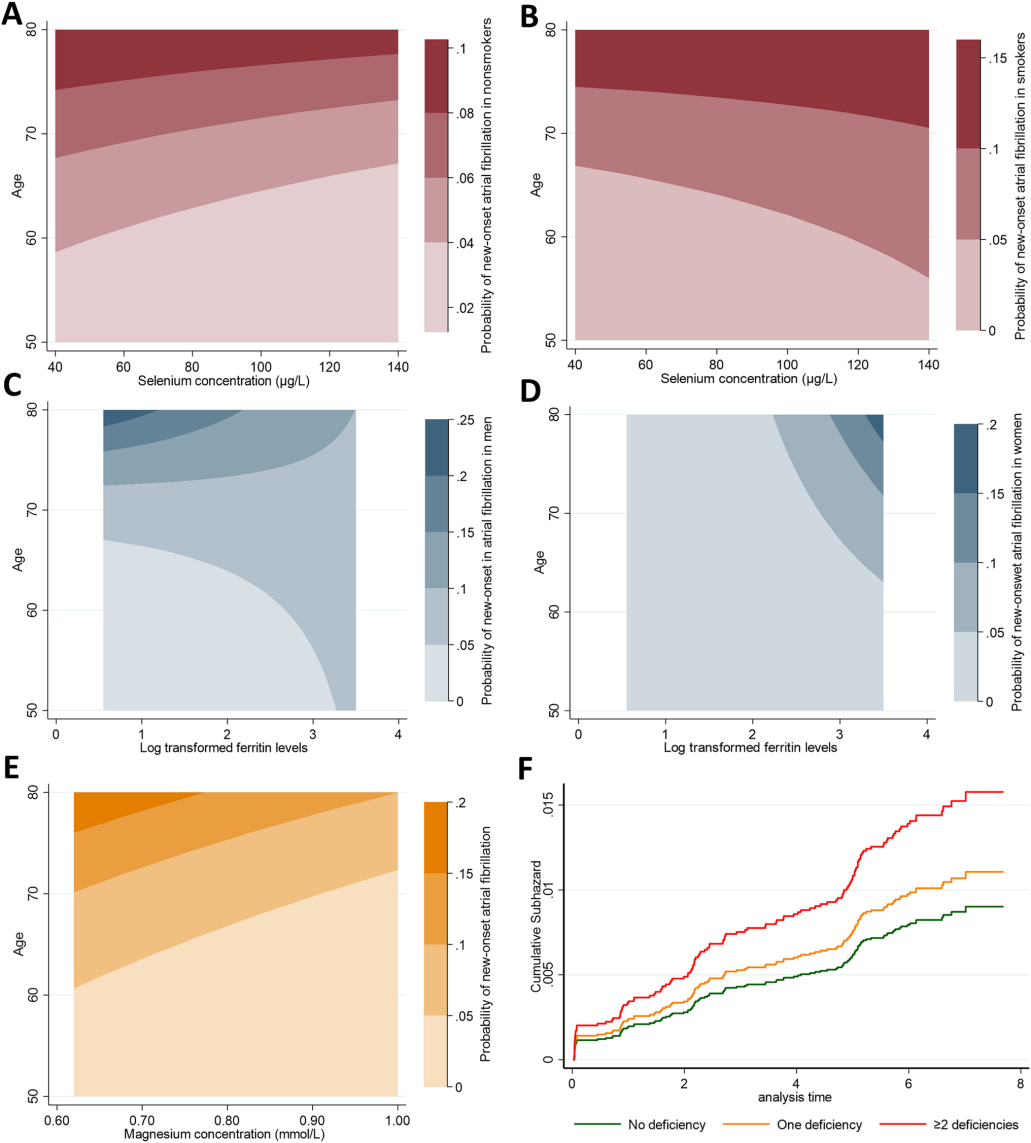
Contour plots of micronutrients concentrations in relation to atrial fibrillation. **A** Contour plot of selenium concentrations in non-smokers. **B** In smokers. **C** Contour plot of ferritin levels in men. **D** In women. **E** Contour plot of magnesium concentrations. **F** A visualization of the association of having one deficiency and two or more deficiencies in relation to new-onset AF

No significant association was found between SeD and new-onset AFib in the uni-variable analysis (hazard ratio (HR) 1.37, 95% confidence interval (CI) 0.93 to 2.02, *p* = 0.109) or after adjusting for the associates of micronutrient deficiencies (HR 1.48, 95% CI 0.97 to 2.24, *p* = 0.069). However, a significant higher risk was observed after adjusting for components of CHARGE-AF model (i.e., model C) (HR 1.51, 95% CI 1.01 to 2.25, *p* = 0.044) (Table 2). There was a significant interaction with smoking status in the uni-variable model as well as model b (*p* = 0.074 and *p* = 0.079, respectively), but not with sex (*p* = 0.462 and *p* = 0.606, respectively) (Table 2). Therefore, we subsequently performed stratified analyses based on smoking status. For non-smoking participants (*N* = 3900), there was a significant association between SeD and new-onset AFib in the uni-variable analysis (HR 1.68, 95% CI 1.09 to 2.59, *p* = 0.018), after adjusting for model B (HR 1.80, 95% CI 1.12 to 2.90, *p* = 0.016), and model C (HR 1.69, 95% CI 1.09 to 2.64, *p* = 0.020). No associations were observed between SeD and new-onset AFib in smoking participants (*N* = 1501; HR 0.74, 95% CI 0.29 to 1.85, *p* = 0.516 in model B; and HR 0.78, 95% CI 0.29 to 2.08, *p* = 0.619 in model C) (Table 3). Considering the competing risk analyses, the direction of effect remained similar and statistically significant in non-smoking participants in all models (Online Tables 5, 6). In addition, as a secondary analysis, we adjusted for interim HF events for the participants who developed HF before AFib (*N* = 10) and the associations remained significant (HR 1.96, 95% CI 1.21 to 3.17, *p* = 0.006 in model C for non-smoking participants) (Online Table S7). Similarly, the associations remained significant in the participants above the age of 60, independent of smoking status (Online Table S8). As a visualization of our findings, the contour plots showed a correlation between lower selenium concentrations and higher risk for new-onset AFib in non-smoking participants, especially for those above 60 (Fig. 2A). An opposite trend was observed in smoking participants (Fig. 2B). After adjustment for components of CHARGE-AF model, restricted cubic splines showed that mainly participants with selenium concentrations < 60–70 μg/L had higher risk to develop AFib (Online Fig. S2A–B).

**Table 2:** Cox proportional hazards regression analyses of each deficiency and the deficiencies combined in association with new-onset atrial fibrillation

Total events (*N* = 136)	Model A						Model B						Model C					
Mean follow-up (= 6.2 years)	HR	CI 95%		*p* value	Interaction with smoking	Interaction with sex	HR	CI 95%		*p* value	Interaction with smoking	Interaction with sex	HR	CI 95%		*p* value	Interaction with smoking	Interaction with sex
Selenium deficiency	1.37	0.93	2.02	0.109	0.074	0.462	1.48	0.97	2.24	0.069	0.079	0.606	1.51	1.01	2.25	0.044	0.106	0.466
Iron deficiency	0.32	0.15	0.69	0.003	Na	0.016	0.69	0.29	1.65	0.408	Na	0.356	0.62	0.25	1.54	0.307	Na	0.209
Magnesium deficiency	1.51	1.02	2.22	0.039	Na	0.885	1.72	1.10	2.69	0.016	Na	0.672	1.40	0.93	2.10	0.110	Na	0.659
Combined deficiencies					Na						Na						Na	
One deficiency	1.51	0.96	2.38	0.077		0.815	1.62	0.96	2.74	0.072		0.588	1.24	0.77	2.00	0.367		0.939
Two or more deficiencies	1.30	0.75	2.23	0.346		0.066	2.11	1.15	3.89	0.017		0.718	1.78	1.02	3.11	0.041		0.381

*eGFR* estimated glomerular filtration rate, *MCV* mean corpuscular volume, *Hs-CRP* high sensitivity C-reactive protein

Model A: univariate analysis. Model B: adjusted for age, sex, BMI, smoking status, glucose, total cholesterol, anemia, kidney function, calcium, potassium and CRP concentrations as well as magnesium and iron deficiencies. Model C: adjusted for Charge-AF model components: age, smoking status, weight, height, systolic blood pressure, the use of antihypertensive drugs, diabetes mellitus, prevalent heart failure, and history of myocardial infarction

**Table 3:** Cox proportional hazards regression analyses of selenium deficiency stratified by smoking status

No. of participants	Non-smoking participants (*N* = 3900)				Smoking participants (*N* = 1501)			
No. of AFib events	*N* = 102				*N* = 34			
Mean follow-up	6.2 years				6.2 years			
	HR	CI 95%		*p* value	HR	CI 95%		*p* value
Model A	1.68	1.09	2.59	0.018	0.75	0.31	1.82	0.524
Model B	1.80	1.12	2.90	0.016	0.74	0.29	1.85	0.516
Model C	1.69	1.09	2.64	0.020	0.78	0.29	2.08	0.619

Models A, B, and C as reported in the text and mentioned under Table 2

### Iron deficiency is not associated with new-onset atrial fibrillation

In participants with ID, we found decreased risk of new-onset AFib in the uni-variable analysis (HR 0.32, 95% CI 0.15 to 0.69, *p* = 0.003), which became non-significant in multivariable analyses (HR 0.62, 95% CI 0.25 to 1.54, *p* = 0.307 in model C) (Table 2). There was a significant interaction with sex in the uni-variable model (*p* = 0.017) but not in multivariable models (Table 2). After sex stratification, no association was found between ID and new-onset AFib in either sex after a multivariable adjustment (Online Table S9). When evaluating older participants separately, participants older than 70 years tended to have higher risks to develop AFib (HR 1.85, 95% CI 0.64 to 5.36, *p* = 0.255 in model C) (Online Table S8). The contour plots and restricted cubic splines showed that men demonstrated a trend of having higher risk to develop AFib at lower ferritin levels, while women had a trend of increased risk only at higher ferritin concentrations (Fig. 2C, D and Online Fig. S2C, D).

### Magnesium deficiency and new-onset atrial fibrillation

Participants with MgD had increased risk of new-onset AFib in the univariate analysis (HR 1.51, 95% CI 1.02 to 2.22, *p* = 0.039) and after adjusting for the associates of the micronutrient deficiencies (HR 1.72, 95% CI 1.10 to 2.69, *p* = 0.016). However, after adjusting for the components of CHARGE-AF model, the increased risk became non-significant (HR 1.40, 95% CI 0.93 to 2.10, *p* = 0.110) (Table 2), as did the sensitivity analysis in participant older than 60 and 70 (Online Table S8) or after correcting for interim HF events (Online Table S7). There were no associations with new-onset AF using the secondary cut-off (< 0.75 mmol/L) for MgD (Online Table S10). The contour plot shows that the risk for new-onset AFib decreases with higher magnesium concentrations (Fig. 2E). The risk for incident AFib was clearly higher in participants with magnesium concentration < 0.85 mmol/L (Online Fig. S2E).

### Participants with two or more deficiencies show increased risk of AFib

There were 2917 (53.5%) participants with one deficiency, 1070 (19.6%) with two deficiencies, 165 (3%) with three deficiencies and 1300 (23.8%) with no deficiency. Participants with two or more deficiencies had higher risk to develop AFib compared to those without deficiencies, regardless of smoking and sex (HR 2.11, 95% CI 1.15 to 3.89, *p* = 0.017 in model B and HR 1.78, 95% CI 1.02 to 3.11, *p* = 0.041 in model C) (Table 2 and Fig. 2F).

## Discussion

We investigated the associations of three micronutrient deficiencies, SeD, ID, and MgD, in relation to new-onset AFib in a large, well-characterized, community-based cohort. After multivariable adjustment for potential confounders, both SeD and MgD were associated with new-onset AFib. However, after adjusting for components of CHARGE-AF model, only SeD in non-smoking participants remained significantly associated with new-onset AFib. Adjusting for interim HF events for the participants who developed HF before AFib strengthened the association with SeD even more. Additionally, we observed a non-significant increased risk in incident AFib in elderly men with ID. These results provide evidence for nutritional imbalances as a new potentially modifiable risk factor in the management and prevention of AFib, independent from HF development.

Our results showed that SeD is associated with a more than 65% increased risk for new-onset AFib in non-smoking participants (Tables 2, 3). Selenium might have particular relevance and mechanistic effects in the cardiomyocytes [8, 12]. Clinically, severe SeD is associated with Keshan disease, a severe dilated cardiomyopathy that is frequently manifested by arrhythmia [28]. In addition, patients with SeD had higher risk of developing post-operative AFib compared to those without SeD [29], which is in line with experimental evidence [30].

Defective mitochondrial Ca^2+^ buffering has been implicated in mitochondrial dysfunction and increased production of ROS. Excessive mitochondrial production of ROS has been implicated in the pathogenesis of HF and AF [31]. As such, cardiomyocytes cultured in the absence of selenium showed increased oxidative stress and reduced ATP production [10]. Moreover, knockdown of type II deiodinase (DIO2), a selenoprotein involved in thyroid hormone metabolism, led to substantial mitochondrial fragmentation, impaired calcium handling, and severely impaired mitochondrial respiration in human cardiomyocytes [32]. Therefore, the low expression of the selenoproteins as a result of SeD might have causative roles in the development of both AFib and HF through mitochondrial dysfunction [8].

We observed no associations between SeD and new-onset AFib in smoking participants. Several studies reported lower levels of selenium in smokers [26, 27]. Cigarettes contain heavy metals (e.g., arsenic, cadmium, nickel, and lead) that are considered important contributors to the health risks and that can interact with selenium [33]. The interactions between heavy metals and selenium might cause changes in the distribution and consumption of selenium and may form toxic complexes, leading to different patterns of selenium effects in smoking participants.

Despite the pathophysiological similarities with SeD, ID was not associated with new-onset AFib. In contrast to selenium, the status of iron is usually determined by measuring of surrogate markers, which might be a limiting factor for a reliable estimation [20]. Ferritin could be a marker for inflammatory status and previous studies showed that participants with high levels of ferritin had increased risk for AFib [34], as do women in association with HF [18], a trend that we observed in our analysis (Fig. 2D). The relevance of ID might be more pronounced in the presence of several comorbidities and not in relatively healthy population as our studied cohort [35]. Of the participants who had ID, 86.8% were women, of which 78% were below the age of 50, indicating the involvement of menstrual bleeding as an important confounder that is likely to mask the association. This hypothesis is supported by the observation that elderly men had higher risk for new-onset AFib (Fig. 2 and Online Table S8), which could be attributed to low iron absorption as well as poor dietary habits [24].

While our data showed that MgD might not be an independent associate for new-onset AFib (Table 2), its potential etiological role cannot be neglected given the increased risk after the adjustment with several confounders [36]. Magnesium is involved in the activity of more than 300 enzymes, has electrophysiological properties and is frequently prescribed as adjunctive treatment to antiarrhythmic drugs [14, 16]. Magnesium homeostasis is complex and not fully understood, partly because most magnesium is stored intracellularly [14]. Consequently, defining the deficiency of this micronutrient can be difficult. There is evidence that indicates that up to 89% of individuals with intracellular magnesium deficiencies might have “normal” serum levels [16]. Nevertheless, our results showed no association with new-onset AFib in individuals with magnesium concentrations < 0.75 mmol/L (Online Table S10 and Online Fig. 2E). This is in contrast to the previously reported study where individuals with magnesium concentrations < 0.77 mmol/L did have increased risk [15]. One likely explanation is the presence of large amount of subjects with the so-called chronic latent magnesium deficiency in our cohort [14, 37]. Patients with this subclinical deficiency do have positive magnesium loading test, the gold standard to diagnose magnesium deficiency, while their plasma magnesium concentrations are between 0.75 and 0.85 mmol/L [14, 37]. This subclinical deficiency is likely to be driven by a low amount of magnesium in the diet, especially considering the increased amount of processed food worldwide [14].

### Strengths and limitations

The studied cohort is a large and a well-characterized community-based cohort with a relatively long follow-up period. This is the first study that investigated three different micronutrient deficiencies in relation to AFib. The participation of large number of relatively young and healthy people is likely to be a limitation in investigating the relevance of ID. Additionally, no Holter monitor or other monitoring methods were utilized to detect AFib in the studied population. These findings are mainly based on observational data and therefore cannot prove causality. In addition, almost all included participants were of Caucasian ethnicity which limits the generalization of data, and no data were available about the intake of proton pump inhibitors.

## Conclusion

In a large community-based cohort, we found that SeD was independently associated with new-onset AFib in non-smoking participants. This association was not observed in smokers, potentially due to mechanistic interactions between heavy metals and selenium. Additionally, there was suggestive increased risk for new-onset AFib in elderly participants with ID, as well as those with subclinical MgD. Interventional studies that investigate the effect of optimizing micronutrients status in a population at risk for developing AFib or in modulating the patterns of AFib are needed, especially in those with SeD.

## Supplementary Information

Below is the link to the electronic supplementary material.

Supplementary file1 (DOCX 672 KB)

## Data Availability

The data that support the findings of this study are available upon a reasonable request from the corresponding author.

## Abbreviations

AFib: Atrial fibrillation
SeD: Selenium deficiency
ID: Iron deficiency
MgD: Magnesium deficiency
HF: Heart failure
