# Supplementary material for: Micronutrient deficiencies and new-onset atrial fibrillation in a community-based cohort: data from PREVEND
Source: Clin Res Cardiol. 2023 Aug 17;114(1):41–52. doi: 10.1007/s00392-023-02276-3 (PMC11772465; doi:10.1007/s00392-023-02276-3)
Supplement: Supplementary file 1 — Supplementary file1 (DOCX 672 KB) [file 392_2023_2276_MOESM1_ESM.docx]

**Supplementary Information**

**Micronutrient deficiencies and new-onset atrial fibrillation in a community-based cohort: Data from PREVEND**

*Brief title: Micronutrients and atrial fibrillation development*

Ali A. Al-Mubarak^1^, Niels Grote Beverborg^1^, Victor Zwartkruis^1^, Colinda van Deutekom^1^, Martin H. de Borst^2^, Ron T. Gansevoort^2^, Stephan J.L. Bakker^2^, Daan J. Touw^3^, Rudolf A. de Boer^1^, Peter van der Meer^1^, Michiel Rienstra^1^, Nils Bomer^1^**^#^**

*^1^Department of Cardiology, University of Groningen, University Medical Center Groningen, Groningen, The Netherlands. ^2^Division of Nephrology, Department of Internal Medicine, University Medical Center Groningen, University of Groningen, Groningen, The Netherlands. ^3^Department of Clinical Pharmacy and Pharmacology, University of Groningen, University Medical Center Groningen, Groningen, The Netherlands*

**^#^Address for correspondence:** Dept. of Cardiology, University Medical Center Groningen, UMCG Post-zone AB43, PO Box 30.001, 9700 RB Groningen, The Netherlands.

Phone +31 50-3615384; E-mail: [n.bomer@umcg.nl](mailto:n.bomer@umcg.nl)


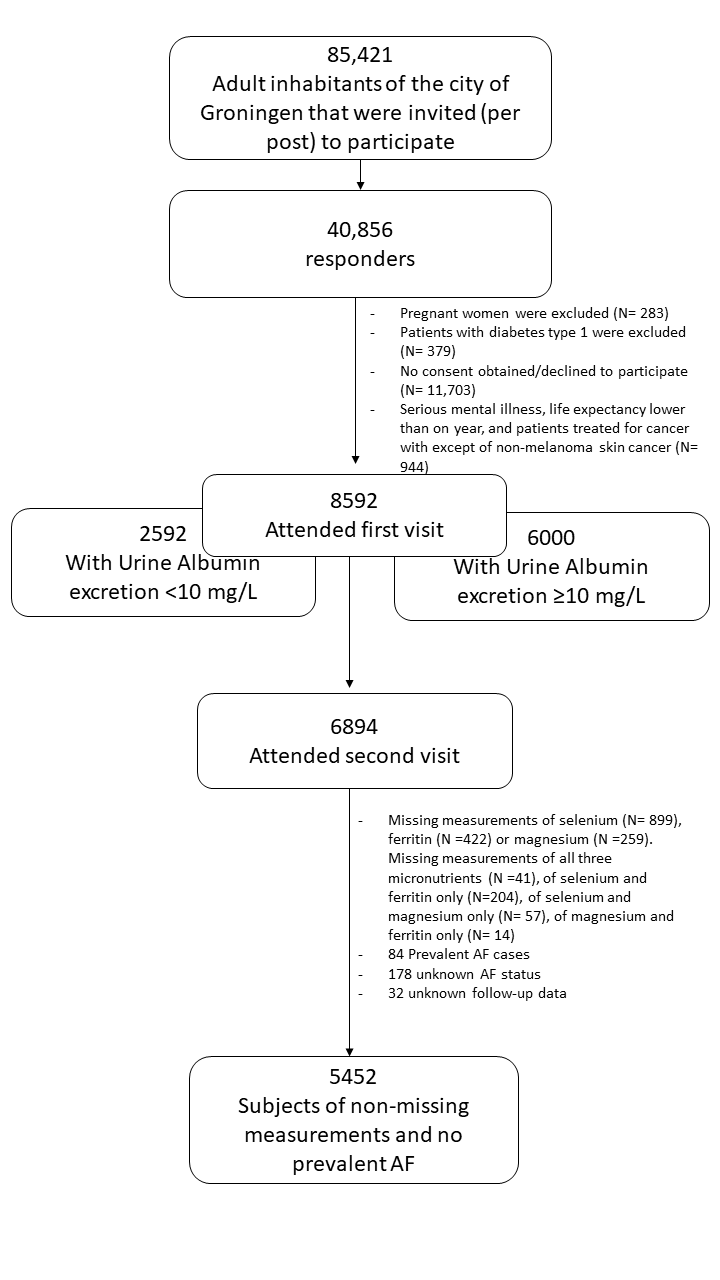


Supplementary Figure 1. The details of the selected cohort.

| **Online Table 1. Associates of iron deficiency** | | | | |
| --- | --- | --- | --- | --- |
|  | Odds ratio | CI 95% | | p value |
| Magnesium deficiency (where 1 is yes) | 1.29 | 1.05 | 1.58 | 0.017 |
| Current smoker (where 1 is yes) | 0.70 | 0.56 | 0.87 | 0.001 |
| Sex (where 1 is woman) | 6.60 | 5.17 | 8.43 | <0.001 |
| Body Mass Index (kg/m²) | 0.97 | 0.94 | 0.99 | 0.01 |
| Plasma calcium (mmol/L) | 0.22 | 0.10 | 0.45 | <0.001 |
| hs-CRP (per doubling) | 0.65 | 0.54 | 0.77 | <0.001 |
| Cholesterol (mmol/L) | 0.85 | 0.77 | 0.94 | 0.001 |
| Age (years) | 0.95 | 0.93 | 0.96 | <0.001 |
| Anemia (where 1 is yes) | 4.55 | 3.55 | 5.84 | <0.001 |
| eGFR (mL/min/1.73 m2) | 0.99 | 0.98 | 1.00 | 0.04 |

| **Online Table 2. Associates** of magnesium deficiency (<85mmol/l) | | | | |
| --- | --- | --- | --- | --- |
|  | Odds ratio | CI 95% | | p value |
| Iron deficiency (where 1 is yes) | 1.39 | 1.15 | 1.68 | 0.001 |
| Body Mass Index (kg/m²) | 1.03 | 1.01 | 1.04 | 0.003 |
| Glucose (mmol/L) | 1.17 | 1.07 | 1.28 | 0.001 |
| Use of antihypertensive medication (where 1 is yes) | 1.24 | 1.04 | 1.49 | 0.017 |
| History of diabetes (where 1 is yes) | 1.67 | 1.09 | 2.54 | 0.018 |
| Systolic blood pressure (mmHg) | 0.99 | 0.99 | 1.00 | <0.001 |
| Cholesterol (mmol/L) | 0.94 | 0.88 | 1.00 | 0.036 |
| Plasma potassium (mmol/L) | 0.46 | 0.37 | 0.58 | <0.001 |
| Urinary albumin excretion (mg/24 h) | 1.00 | 1.00 | 1.00 | 0.016 |
| eGFR (mL/min/1.73 m2) | 1.01 | 1.00 | 1.01 | <0.001 |

| **Online Table 3. Number of subjects with missing values per variable** | |
| --- | --- |
|  | N |
| Age | 0 |
| Sex | 0 |
| Weight | 1 |
| Height | 0 |
| Body Mass Index | 1 |
| Systolic blood pressure | 1 |
| Current smoker | 51 |
| Plasma potassium | 0 |
| Plasma calcium | 2 |
| Glucose | 91 |
| Cholesterol | 28 |
| Anemia | 27 |
| hs-CRP | 329 |
| eGFR | 265 |
| Use of antihypertensive medication | 9 |
| History of diabetes mellitus | 101 |
| History of myocardial infarction | 105 |
| History of heart failure | 0 |

| **Online Table 4. Participant characteristics based on magnesium status (secondary cutoff)** | | | | |
| --- | --- | --- | --- | --- |
|  | All subjects | No Magnesium deficiency (≥75mmol/L) | Magnesium deficiency (<75mmol/L) | p-value |
| N | 5452 | 5015 | 437 |  |
| Plasma magnesium (mmol/L) | 0.82 (0.06) | 0.83 (0.06) | 0.71 (0.03) | <0.001 |
| Age (years) | 53.3 (12.0) | 53.2 (11.9) | 53.7 (12.4) | 0.40 |
| Women (%) | 2843 (52.1%) | 2591 (51.7%) | 252 (57.7%) | 0.016 |
| Body Mass Index (kg/m²) | 26.6 (4.3) | 26.5 (4.2) | 27.8 (5.0) | <0.001 |
| Systolic blood pressure (mmHg) | 125.6 (18.7) | 125.4 (18.6) | 128.1 (20.1) | 0.003 |
| Current smoker (%) | 1501 (27.8%) | 1373 (27.6%) | 128 (29.7%) | 0.36 |
| History of Myocardial infarction (%) | 333 (6.2%) | 294 (6.0%) | 39 (9.1%) | 0.010 |
| History of diabetes (%) | 317 (5.9%) | 237 (4.8%) | 80 (18.5%) | <0.001 |
| History of CVA (%) | 60 (1.1%) | 51 (1.0%) | 9 (2.1%) | 0.044 |
| History of HF (%) | 47 (0.9%) | 33 (0.7%) | 14 (3.2%) | <0.001 |
| Glucose (mmol/L) | 5.0 (1.2) | 5.0 (1.1) | 5.6 (2.0) | <0.001 |
| Cholesterol (mmol/L) | 5.4 (1.1) | 5.4 (1.1) | 5.3 (1.1) | 0.021 |
| eGFR (mL/min/1.73 m2) | 92.2 (16.8) | 92.1 (16.7) | 92.6 (18.2) | 0.59 |
| Kidney function below 60 ml/min/m^2^ (%) | 209 (4.0%) | 182 (3.8%) | 27 (6.4%) | 0.009 |
| Urinary albumin excretion (mg/24 h) | 8.0 (5.9, 13.0) | 7.9 (5.9, 12.7) | 9.6 (6.0, 19.6) | <0.001 |
| Hemoglobin (mmol/L) | 8.5 (0.8) | 8.5 (0.8) | 8.3 (0.8) | <0.001 |
| Anemia (%) | 514 (9.5%) | 445 (8.9%) | 69 (15.9%) | <0.001 |
| MCV (fL) | 90.5 (4.5) | 90.5 (4.5) | 90.6 (5.0) | 0.61 |
| Hematocrit (v/v) | 0.4 (0.0) | 0.4 (0.0) | 0.4 (0.0) | <0.001 |
| Serum iron (umol/L) | 15.9 (5.7) | 15.9 (5.6) | 15.6 (5.9) | 0.25 |
| Transferrin (g/L) | 2.6 (0.4) | 2.6 (0.4) | 2.6 (0.4) | 0.55 |
| Transferrin saturation (%) | 151.2 (71.9, 280.3) | 151.2 (72.7, 278.6) | 151.5 (68.2, 294.5) | 0.77 |
| Ferritin (ug/L) | 96.0 (47.5, 172.0) | 96.0 (48.0, 171.0) | 98.0 (45.0, 188.0) | 0.80 |
| Iron deficiency (%) | 797 (14.6%) | 722 (14.4%) | 75 (17.2%) | 0.12 |
| Selenium concentration (µg/L) | 84.5 (19.2) | 84.5 (19.3) | 83.9 (17.7) | 0.52 |
| Selenium deficiency (%) | 1155 (21.2%) | 1065 (21.2%) | 90 (20.6%) | 0.75 |
| Plasma calcium (mmol/L) | 2.3 (0.1) | 2.3 (0.1) | 2.3 (0.1) | <0.001 |
| Plasma potassium (mmol/L) | 4.2 (0.3) | 4.2 (0.3) | 4.1 (0.3) | <0.001 |
| hs-CRP (mg/L) | 1.3 (0.6, 2.9) | 1.3 (0.6, 2.9) | 1.6 (0.7, 3.6) | 0.006 |
| Antihypertensive medication | 1176 (21.6%) | 1019 (20.4%) | 157 (35.9%) | <0.001 |

| Online Table 5. Competing risk analyses of each deficiency and the deficiencies combined in association with new-onset atrial fibrillation. | | | | | | | | | | | | | | | | | | | | | | |
| --- | --- | --- | --- | --- | --- | --- | --- | --- | --- | --- | --- | --- | --- | --- | --- | --- | --- | --- | --- | --- | --- | --- |
| Total AF events (N =136), competing risks events (N=185) | Model a | | | | | | Model b | | | | | | | Model c | | | | | | | | |
| Mean follow-up (=6.2 years) | SHR | CI 95% | | p value | Interaction with smoking | Interaction with sex | SHR | CI 95% | | p value | | Interaction with smoking | Interaction with sex | SHR | CI 95% | | | p value | | Interaction with smoking | Interaction with sex |  |
| Selenium Deficiency | 1.37 | 0.93 | 2.01 | 0.110 | 0.075 | 0.464 | 1.49 | 0.97 | 2.29 | | 0.070 | 0.081 | 0.628 | 1.49 | 1.00 | 2.22 | 0.053 | | 0.084 | | 0.514 |  |
| Iron deficiency | 0.32 | 0.15 | 0.70 | 0.004 |  | 0.017 | 0.70 | 0.30 | 1.64 | | 0.409 |  | 0.322 | 0.59 | 0.23 | 1.49 | 0.266 | |  | | 0.244 |  |
| Magnesium deficiency | 1.50 | 1.01 | 2.20 | 0.042 |  | 0.899 | 1.71 | 1.09 | 2.69 | | 0.020 |  | 0.686 | 1.40 | 0.93 | 2.12 | 0.108 | |  | | 0.675 |  |
| Combined deficiencies |  |  |  |  |  |  |  |  |  | |  |  |  |  |  |  |  | |  | |  |  |
| One deficiency | 1.49 | 0.95 | 2.36 | 0.084 |  | 0.824 | 1.61 | 0.94 | 2.74 | | 0.080 |  | 0.606 | 1.23 | 0.76 | 1.99 | 0.408 | |  | | 0.958 |  |
| Two or more deficiencies | 1.29 | 0.75 | 2.23 | 0.352 |  | 0.07 | 2.11 | 1.13 | 3.94 | | 0.019 |  | 0.708 | 1.75 | 1.00 | 3.06 | 0.050 | |  | | 0.415 |  |

Model a: univariate analysis. Model b: adjusted for age, sex, BMI, smoking status, glucose, total cholesterol, anemia, kidney function, calcium, potassium and CRP concentrations as well as magnesium and iron deficiencies. Model c: adjusted for Charge-AF model components: age, smoking status, weight, height, systolic blood pressure, the use of antihypertensive drugs, diabetes mellitus, prevalent heart failure, and history of myocardial infarction.

| **Online Table 6. Competing risk analyses of selenium deficiency stratified by smoking status** | | | | | | | | |
| --- | --- | --- | --- | --- | --- | --- | --- | --- |
| N of participants | Non-smoking participants (N=3900) | | | | Smoking participants (N=1501) | | | |
| N of AFib events | N=102 | | | | N=34 | | | |
| N of competing events | N=125 | | | | N=57 | | | |
| Mean follow-up | 6.2 years | | | | 6.2 years | | | |
|  | **HR** | **CI 95%** | | **p value** | **HR** | **CI 95%** | | **p value** |
| model a | 1.68 | 1.09 | 2.58 | 0.019 | 0.75 | 0.31 | 1.82 | 0.523 |
| model b | 1.82 | 1.11 | 2.97 | 0.017 | 0.76 | 0.29 | 1.98 | 0.575 |
| model c | 1.69 | 1.08 | 2.66 | 0.022 | 0.71 | 0.27 | 1.90 | 0.494 |

Models a, b and c as reported in the text and mentioned under Online Table 5.

| Online Table 7. Cox proportional hazards regression analyses of each deficiency after adjusting for interim HF events for participants who developed HF before AFib (adjustment included model C variables) | | | | | |
| --- | --- | --- | --- | --- | --- |
|  | N of AF events | HR | CI 95% | | p value |
| Selenium deficiency |  |  |  |  |  |
| Total cohort (N=5452) | 124 | 1.62 | 1.05 | 2.50 | 0.029 |
| Non-smokers only (N=3900) | 92 | 1.96 | 1.21 | 3.17 | 0.006 |
| Smokers (N=1501) | 32 | 0.74 | 0.25 | 2.19 | 0.586 |
| Iron deficiency | 124 | 0.77 | 0.31 | 1.92 | 0.580 |
| Magnesium deficiency | 124 | 1.43 | 0.93 | 2.18 | 0.101 |

| Online Table 8. Cox proportional hazards regression analyses of each deficiency in association with new-onset AFib in participants older than 60 years-old and participants older than 70 years-old | | | | | | | | | |
| --- | --- | --- | --- | --- | --- | --- | --- | --- | --- |
| N of participants |  | Above the age of 60 (N =1582) | | | | Above the age of 70 (N =621) | | | |
| N of AFib events | N=96 | | | | | N=45 | | | |
| Mean follow-up | 5.97 years | | | | | 5.76 years | | | |
|  | model | HR | CI 95% | | p value | HR | CI 95% | | p value |
| Selenium deficiency | a | 1.72 | 1.10 | 2.67 | 0.017 | 1.79 | 0.93 | 3.44 | 0.079 |
|  | b | 1.75 | 1.07 | 2.86 | 0.025 | 2.05 | 0.99 | 4.23 | 0.052 |
|  | c | 1.80 | 1.14 | 2.84 | 0.011 | 1.94 | 0.98 | 3.85 | 0.057 |
| Iron deficiency | a | 0.97 | 0.39 | 2.39 | 0.948 | 1.49 | 0.53 | 4.15 | 0.449 |
|  | b | 0.91 | 0.32 | 2.59 | 0.865 | 2.33 | 0.65 | 8.36 | 0.195 |
|  | c | 1.15 | 0.46 | 2.85 | 0.770 | 1.85 | 0.64 | 5.36 | 0.255 |
| Magnesium deficiency | a | 1.47 | 0.94 | 2.30 | 0.094 | 1.60 | 0.81 | 3.18 | 0.180 |
|  | b | 1.61 | 0.95 | 2.71 | 0.077 | 1.23 | 0.57 | 2.62 | 0.598 |
|  | c | 1.19 | 0.75 | 1.90 | 0.456 | 1.22 | 0.59 | 2.51 | 0.586 |

Models a, b and c as reported in the text and mentioned under Online Table 5.


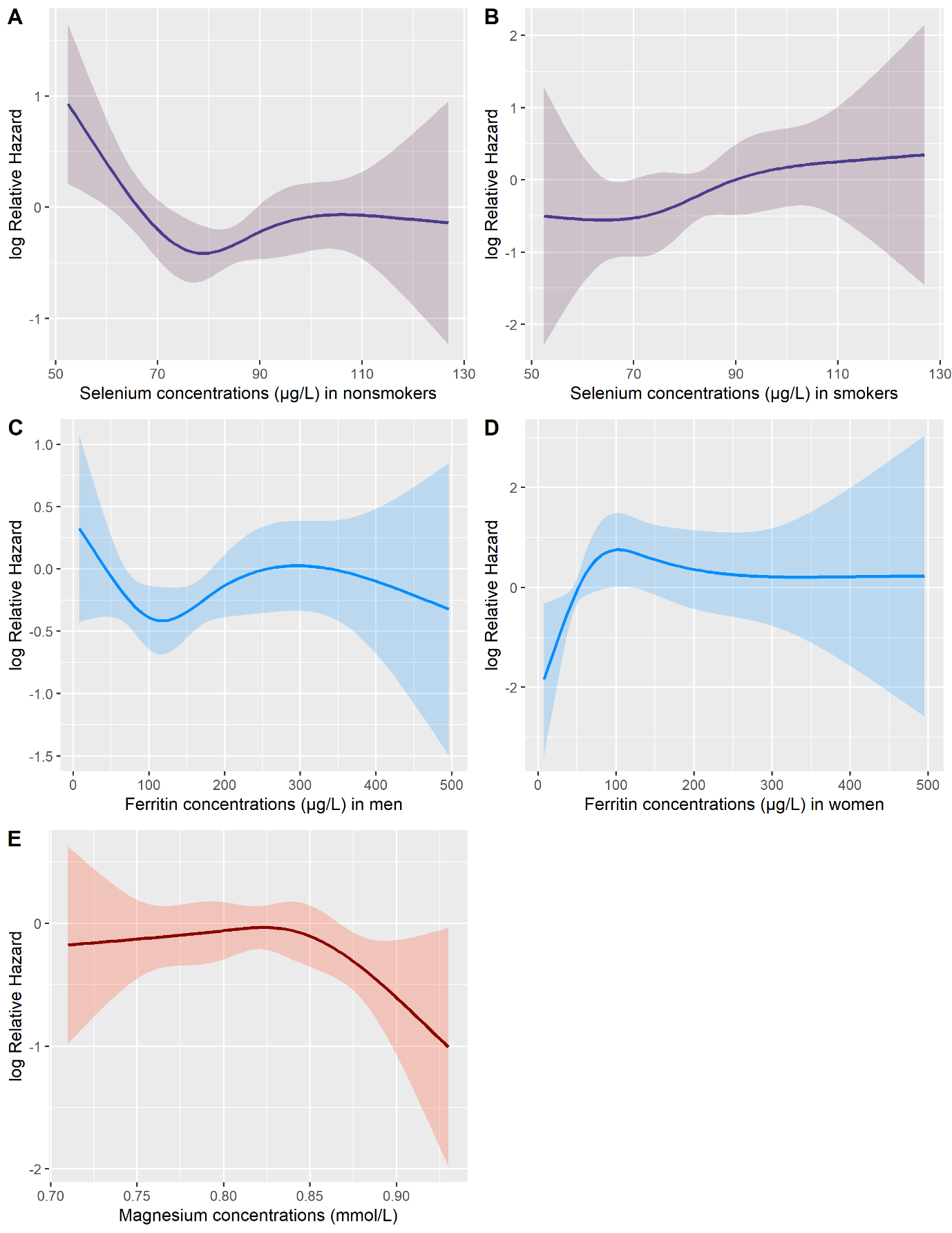


Online Figure 2. Restricted cubic splines of the three parameters adjusted for components of CHARGE-AF model. A. Selenium in nonsmokers. B. Selenium in smokers. C. Ferritin in men. D. Ferritin in women. E. Magnesium.

| Online Table 9. Cox proportional hazards regression analyses of selenium deficiency stratified by smoking status | | | | | | | | |
| --- | --- | --- | --- | --- | --- | --- | --- | --- |
| N of participants | Men (N=2609) | | | | Women (N=2843) | | | |
| N of AFib events | N=97 | | | | N=39 | | | |
| Mean follow-up | 6.12 years | | | | 6.28 years | | | |
|  | HR | CI 95% | | p value | HR | CI 95% | | p value |
| model a | 1.33 | 0.54 | 3.28 | 0.530 | 0.17 | 0.04 | 0.69 | 0.013 |
| model b | 0.92 | 0.31 | 2.69 | 0.879 | 0.50 | 0.11 | 2.24 | 0.362 |
| model c | 1.22 | 0.49 | 3.02 | 0.672 | 0.48 | 0.11 | 2.08 | 0.328 |

Models a, b and c as reported in the text and mentioned under Online Table 5.

| Online Table 10. Cox proportional hazards regression analyses of the secondary cut-off of magnesium deficiency (<0.75mmol/L) | | | | |
| --- | --- | --- | --- | --- |
| N of AFib events (=136) | Magnesium deficiency (<0.75mmol/L) | | | |
|  | HR | CI 95% | | p value |
| model a | 1.20 | 0.68 | 2.11 | 0.529 |
| model b | 1.14 | 0.63 | 2.08 | 0.670 |
| model c | 0.85 | 0.47 | 1.55 | 0.600 |

Models a, b and c as reported in the text and mentioned under Online Table 5.
